# Extended neuromonitoring in aortic arch surgery: A case series

**DOI:** 10.1007/s00101-021-00983-y

**Published:** 2021-06-07

**Authors:** Marcus Thudium, Evgeniya Kornilov, Tobias Hilbert, Mark Coburn, Christopher Gestrich

**Affiliations:** 1 Department of Anesthesiology and Intensive Care Medicine, University Hospital Bonn, Venusberg Campus 1, 53127 Bonn, Germany; 2 Weizmann Institute of Science, Rehovot, Israel; 3 Department of Cardiothoracic Surgery, University Hospital Bonn, Bonn, Germany

**Keywords:** Aortic dissection, Transcranial doppler, NIRS, EEG, Cerebral perfusion, Aortendissektion, Transkranieller Doppler, NIRS, EEG, Hirnperfusion

## Abstract

**Background:**

Aortic arch repair for aortic dissection is still associated with a high mortality rate. Providing adequate means of neuromonitoring to guide cerebral hemodynamics is advantageous, especially during selective anterior cerebral perfusion (SACP).

**Objective:**

We aimed to investigate an easy multimodal neuromonitoring set-up consisting of processed electroencephalography (EEG), near infrared spectroscopy (NIRS), and transcranial doppler sonography (TCD).

**Material and methods:**

We collected intraoperative data from six patients undergoing surgery for aortic dissection. In addition to standard hemodynamic monitoring, patients underwent continuous bilateral NIRS, processed EEG with bispectral index (BIS), and intermittent transcranial doppler sonography of the medial cerebral artery (MCA) with a standard B‑mode ultrasound device. Doppler measurements were taken bilaterally before cardiopulmonary bypass (CPB), during CPB, and during SACP at regular intervals.

**Results:**

Of the patients four survived without neurological deficits while two suffered fatal outcomes. Of the survivors two suffered from transient postoperative delirium. Multimodal monitoring led to a change in CPB flow or cannula repositioning in three patients. Left-sided mean flow velocities of the MCA decreased during SACP, as did BIS values.

**Conclusion:**

Monitoring consisting of BIS, NIRS, and TCD may have an impact on hemodynamic management in aortic arch operations.

**Supplementary Information:**

The online version of this paper (10.1007/s00101-021-00983-y) contains a supplementary table showing all intraoperative measurements, which is available to authorized users.

Article and supplementary material are available at www.springermedizin.de. Please enter the title of the article in the search field, the additional material can be found at the article under “Ergänzende Inhalte”.

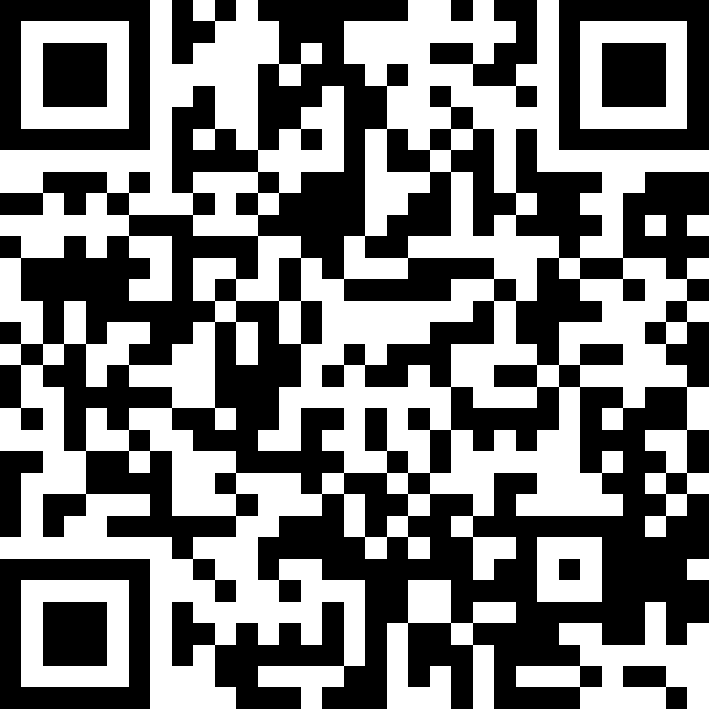

## Abstract (de)

**Hintergrund:**

Operationen am Aortenbogen bei Aortendissektion sind immer noch mit einer hohen Sterblichkeit verbunden. Vorteilhaft wäre hierbei ein adäquates Neuromonitoring zur Steuerung der zerebralen Hämodynamik, insbesondere während der Phase der selektiven Hirnperfusion (SACP).

**Fragestellung:**

Wir untersuchten ein einfaches multimodales Neuromonitoring-Setup, bestehend aus Elektroenzephalographie (EEG), Nahinfrarotspektroskopie (NIRS) und transkranieller Dopplersonographie (TCD).

**Material und Methoden:**

Sechs Patienten mit Operationen des Aortenbogens aufgrund einer Aortendissektion wurden eingeschlossen. Zusätzlich zum hämodynamischen Standardmonitoring erhielten diese eine kontinuierliche bilaterale NIRS-Ableitung, eine prozessierte EEG mit bispektralem Index (BIS) und eine intermittierende transkranielle Dopplersonographie der A. cerebri media (MCA) mit einem Standard-B-Mode-Ultraschallgerät. Dopplermessungen wurden beidseits in regelmäßigen Intervallen vor und während des kardiopulmonalen Bypasses (CPB) sowie während SACP durchgeführt.

**Ergebnisse:**

Vier Patienten überlebten ohne neurologisches Defizit, während zwei verstarben. Zwei der Überlebenden erlitten ein vorübergehendes postoperatives Delir. Multimodales Monitoring resultierte in einer Veränderung des CPB-Flusses oder einer Kanülenrepositionierung bei drei Patienten. Die linksseitige Flussgeschwindigkeiten der MCA wie auch die BIS-Werte waren während SACP geringer.

**Schlussfolgerung:**

Ein Monitoring bestehend aus BIS, NIRS und TCD kann das hämodynamische Management während Operationen des Aortenbogens beeinflussen.

## Infobox 1 Treten Sie in den Austausch

Diese Arbeit wurde für *Der Anaesthesist* in Englisch eingereicht und angenommen. Die deutsche Zusammenfassung wurde daher etwas ausführlicher gestaltet. Wenn Sie über diese Zusammenfassung hinaus Fragen haben und mehr wissen wollen, nehmen Sie gern in Deutsch über die Korrespondenzadresse am Ende des Beitrags Kontakt auf. Die Autor*innen freuen sich auf den Austausch mit Ihnen.

## Introduction and background

Aortic arch repair is one of the most invasive procedures currently performed. It requires phases of circulatory arrest and selective anterior cerebral perfusion (SACP) via the carotid arteries [1]. Cerebral malperfusion and brain injury are feared complications. In SACP, few neuromonitoring options remain, such as near infrared spectroscopy (NIRS) and transcranial doppler sonography (TCD). While NIRS has become the standard of care in aortic arch repair the TCD is rarely utilized. We hypothesized that a multimodal approach could be advantageous since it provides a more comprehensive feedback to guide cerebral perfusion [2, 3]. We report on the use of an easy monitoring set-up implemented in six patients.

## Study design and investigation methods

This manuscript adheres to the guidelines for case reports (CARE) statement. The series was approved by the ethics committee of the University of Bonn (No 353/16). Informed consent was waived due to the observational nature of the study.

Before induction of anesthesia patients received standard monitoring consisting of an electrocardiogram, peripheral oxygen saturation and arterial cannulation of the left radial artery for continuous blood pressure measurement. Induction was performed with etomidate and sufentanil. Patients were orotracheally intubated and received central venous catheterization as well as a urinary catheter.

The arterial cannula for the cardiopulmonary bypass (CPB) was inserted directly into the right subclavian artery according to the standard protocol. The subclavian artery was accessed by an incision caudal of the right clavicle. Patients received median sternotomy and were injected with heparin 400 IU/kg bodyweight. The venous two-staged cannula was inserted via the right atrium. During CPB, patients were cooled to 25–28 °C and the aorta was cross-clamped. Acid-base management was performed using the alpha-stat method. To inspect and repair the aortic arch, a short period of complete circulatory arrest was accepted, the brachiocephalic artery was then clamped and selective cerebral perfusion was started at 10 ml/kg per minute. A second balloon-tipped cannula was inserted into the left carotid artery to ensure sufficient blood flow to both hemispheres. The use of pharmacological neuroprotection was left to the decision of the anesthesia/surgical team, resulting in the application of 500 mg thiopental and 500 mg prednisolone in all patients, as is commonly practiced in this setting [4].

Prior to skin incision, NIRS optodes were attached bilaterally to the patient’s forehead, and NIRS was measured continuously. Next to the NIRS optodes, a bispectral index (BIS) electrode was attached, and BIS was also measured continuously (BIS Vista, Medtronic, Dublin, Ireland).

For TCD measurements, a S5‑1 transducer on a CX 50 ultrasound machine (Philips Health Systems, Hamburg, Germany) was used. To achieve sonographic access to the medial cerebral artery (MCA), the ultrasound transducer was placed on the temporal fossa above the zygomatic arch and the MCA was located with color doppler. The MCA velocity (MCAV) was measured with pulsed wave doppler in a depth of 3.5–4.5 cm where there was little or no angulation between the direction of the ultrasound waves and direction of blood flow. The TCD measurements were obtained on both sides of the skull after skin incision, subsequent to beginning of CPB, and in 10 min intervals during SACP. To reduce interrater variations, all measurements were taken by one investigator experienced in TCD.

An MCAV reduction of > 50% from baseline or a relative decrease in NIRS-derived cerebral oxygen saturation (rSO_2_) of more than 20% from baseline values were considered to represent malperfusion with the need for hemodynamic adjustment.

## Results

### Case descriptions

Between January 2018 and March 2019, a total of 6 consecutive patients with aortic dissection underwent aortic arch repair surgery.

Patient 1 was operated on due to a retrograde dissection months after an endovascular stent graft repair (thoracic endovascular aortic repair, TEVAR) of a type B aortic dissection. During SACP, regional cerebral oxygen saturation (rSO_2_) showed a right-sided and left-sided reduction of 5% and 2% (6.3% and 3.5% relative reduction), respectively. The TCD showed a right-sided increase in MCAV of 3 cm/s (16%) while left-sided MCAV decreased by 5 cm/s (23%), which was rated as stable perfusion. The patient recovered well and showed no signs of neurological disorders.

In patient 2 operative conditions were difficult due to extreme obesity. This patient was also operated on as redo surgery after retrograde dissection shortly after TEVAR due to type B aortic dissection. During beginning of SACP the rSO_2_ and MCAV dropped drastically. Therefore, cannulation was altered and blood flow over the CPB was increased. This only resulted in an insufficient increase in values, whereas right-sided NIRS and TCD values decreased by 22% (33% relative reduction) and 27 cm/s (63%) and left side NIRS and TCD values decreased by 7% (10% relative reduction) and 8 cm/s (19%), respectively. After prolonged CPB time, weaning proved unsuccessful because of right ventricular failure and extracorporeal life support had to be established. In the postoperative course, the patient developed sepsis with subsequent multiorgan failure and died on the intensive care unit (ICU).

In patient 3 there was a decrease in left-sided rSO_2_ values by 8% (11% relative decrease) during SACP while the right side was stable. The TCD showed a right-sided and left-sided reduction in MCAV by 29 cm/s (45%) and 19 cm/s (45%), respectively. We decided to increase the blood flow, tolerated higher NIRS values and used TCD for further references. After successful surgery the patient recovered well.

In patient 4 there was an increase in right and left rSO_2_ during SACP by 16% and 9% (20% and 11% relative increase), respectively. Conversely, there was a reduction of left-sided MCAV by 18 cm/s (53%) while the right side increased by 4 cm/s (15%). Since SACP flow was high at this time (1.4 L/min) and cannula position was correct, we saw no possibility of hemodynamic improvement. This patient showed prolonged postoperative delirium.

In patient 5 right frontal rSO_2_ was low at the time of skin incision. After standard cannulation and during SACP, right-sided and left-sided rSO_2_ increased by 11% and 9% (21% and 17% relative increase), respectively. The TCD showed no relevant differences in MCAV on the right side but revealed a decrease on the left side by 40 cm/s (50%). Despite this decrease, we accepted these values after verifying the cannula position since left MCAV after skin incision was exceptionally high (82 cm/s), decreasing to 42 cm/s with initiation of CPB. The patient recovered well from surgery but showed postoperative hyperactive delirium, which was additionally attributable to the patient’s long history of i.v. substance abuse.

In patient 6 conditions for surgery were adverse and the distal end of the aortic prothesis could not be attached without redissection. Since SACP time was already prolonged, there was consensus that there was no remaining option for this patient, so death in tabula was the final result. In this patient, NIRS was reduced during SACP by 15% and 11% (20% and 12% relative decrease) on the right and left sides, respectively, while TCD measurements showed a severe reduction of mean blood flow velocity during SACP by 27 cm/s (67%) and 12 cm/s (51%) on the right and left sides, respectively. The SACP cannulae had to be adjusted repeatedly after compromised flow was seen in TCD.

**Fig. 1:**
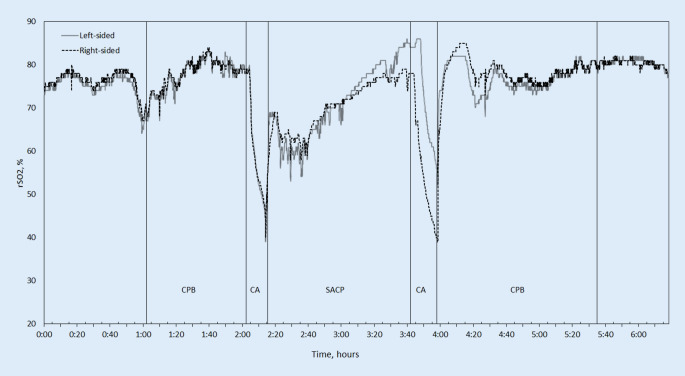
Representative example of NIRS measurement during aortic arch repair procedure with corresponding operation phases (patient 1). *rSO*_*2*_ regional cerebral oxygen saturation, *CPB* cardiopulmonary bypass, *CA* circulatory arrest, *SACP* selective antegrade cerebral perfusion

Baseline characteristics of the patients are summarized in Table 1. A representative graph of rSO_2_ during surgery is shown in Fig. 1. In an overview of all patients, MCAV as well as rSO_2_ decreased during SACP (see Table 2, see supplementary material).

**Table 1:** Patient baseline characteristics. Data presented as median (range) or number (%)

Parameter	Incidence
Age, years	64 (54.5–79.5)
Male sex, *n*	3 (50)
Body mass index	22 (21.3–41.3)
Body surface area, m^2^	1.88 (1.8–2.53)
Delirium, *n*	2 (33.3)
Death, *n*	2 (33.3)
Bypass time, h	4.67 (3.27–6.23)
Cross-clamp time, h	3.02 (2.08–3.25)

**Table 2:** Intraoperative BIS, NIRS, and MCAV values of all patients (survivors and non-survivors). Data presented as median (range)

Parameter	Before CPB	During SCAP
*All patients (n* *=* *6)*		
NIRS right frontal, %	72.3 (52.0–79.0)	66.8 (44.0–95.0)
NIRS left frontal, %	72.9 (52.0–87.0)	67.5 (59.0–92.0)
MCAV right-sided, cm/s	35.5 (18.4–64.0)	25.0 (12.9–36.7)
MCAV left-sided, cm/s	35.1 (21.3–82.0)	20.2 (11.3–41.1)
BIS	38.5 (35.0–40.0)	4.4 (0.0–18.0)
*Survivors (n* *=* *4)*		
NIRS right frontal, %	72.3 (52.0–79.0)	70.6 (63.0–95.0)
NIRS left frontal, %	72.9 (52.0–87.0)	67.5 (61.0–92.0)
MCAV right-sided, cm/s	31.6 (18.4–64.0)	31.7 (21.3–36.7)
MCAV left-sided, cm/s	38.9 (21.3–82.0)	20.2 (16.0–41.1)
BIS	38.5 (35.0–40.0)	9.5 (2.0–18.0)
*Non-survivors (n* *=* *2)*		
NIRS right frontal, %	71.5 (66.0–77.0)	52.9 (44.0–61.8)
NIRS left frontal, %	76.5 (66.0–87.0)	67.7 (59.0–76.3)
MCAV right-sided, cm/s	40.5 (39.0–42.0)	14.1 (12.8–15.3)
MCAV left-sided, cm/s	29.5 (23.0–36.0)	20.1 (11.3–29.0)
BIS	38.0 (36.0–40.0)	2.9 (0.0–5.8)

*NIRS* near infrared spectroscopy, *MCAV* middle cerebral artery mean flow velocity, *BIS* bispectral index, *CPB* cardiopulmonary bypass, *SCAP* selective anterior cerebral perfusion

In three patients (patients 2, 3, and 6) CPB flow was adjusted or carotid cannulae were repositioned after TCD measurements. A low MCA blood flow signal intensity on the color doppler image also proved to be an important indicator for cannula repositioning in patients 2, 3, and 6.

## Discussion

This case series revealed that BIS, rSO_2_, and MCAV were preserved before and during CPB. During SACP the average rSO_2_ was slightly decreased while the average MCAV indicated possible hypoperfusion. The BIS showed only minimal cerebral electrical activity during SACP.

Inadequate cerebral perfusion during SACP can result in unfavorable neurological outcome. The best parameters to monitor adequate cerebral perfusion are still unclear. Currently, the use of NIRS as a simple and continuous monitoring strategy seems to have become the standard of care and is currently recommended [5]; however, the validity of the method is still being disputed. During CPB a dislocation of the arterial cannula may be noticed with NIRS monitoring [6]. Since possible poor cerebral oxygenation as indicated by NIRS may result from several different factors, a protocol in cases of desaturation has been introduced by Denault et al. [7]. Joshi et al. even used NIRS to predict limits of cerebral autoregulation [8]. In our series, we show some major differences in cerebral oxygenation during deep hypothermic cardiac arrest (Fig. 1); however, the method also has its limits. In patients with brain atrophy, the validity of rSO_2_ is questionable [5, 9]. Extracerebral contamination of the NIRS signal has also been shown as well as a limited comparability of different devices [10, 11]. Additionally, rSO_2_ is affected by variations in hemoglobin concentration [9].

The TCD has long been used to assess cerebral hemodynamics [12]. There is generally a consensus that TCD can detect changes in cerebral blood flow [13]. In our patients we noticed that pre-CPB the MCAV was reduced, possibly due to the dissection. Alternatively, increased MCAV values during CPB could represent hyperperfusion, which we described previously [14]. Wang et al. provided some suggestions on sufficient cerebral blood flow and reported a close correlation between NIRS and TCD measurements [15]. In our patients, MCAV, unlike NIRS, indicated possible hypoperfusion during SACP. Whether this can be explained by a higher sensitivity of TCD regarding hemodynamic changes or conversely indicate an effective neuroprotection strategy remains unclear. For continuous measurement, a headframe with Doppler probes could represent the ideal monitoring tool. This has been presented by Estrera et al. but with a neurosonographer since this method requires intense training, which is also the reason why TCD is not generally recommended [5, 16]. In a report by Ghazy et al. the B‑mode transducer was fixed with a movable arm [3]. We accepted the disadvantage of noncontinuous TCD measurements in trade-off for bilateral TCD measurements. Denault et al. recently proposed a modified NIRS algorithm in which ultrasound imaging plays a distinct role in hemodynamic evaluation and cerebral imaging in the case of increased intracranial pressure [17].

We propose that ultrasound may also be used to gain additional information about cerebral hemodynamics during SACP. Unfortunately, it remains unknown how to determine the individual adequate cerebral blood flow with TCD, since basal arteries have been shown to undergo caliber changes [18]. Different vessel diameters can also result in difficult intersubject comparability. Another cause for misleading TCD measurements is the susceptibility to high or low arterial CO_2_ tension, resulting in cerebral vasodilation in hypercapnia and vasoconstriction in hypocapnia and therefore in decreased or increased flow velocities in TCD [19]. In the case of hypoxia, vasodilation has been shown to occur using color doppler ultrasound [20]. We did not assess MCA diameter in our patients. It must also be noted that stable frontal NIRS measurements may still occur even if MCA flow is compromised on the ipsilateral side since the anterior cerebral artery (ACA) may be supplied by the anterior communicating artery from the other hemisphere. This may also explain some of the inconsistencies between measurements using NIRS and TCD. As an alternative, NIRS of the MCA territory with combined TCD of the MCA would be an option, as the MCA supplies the largest regions of the brain and is therefore most important to monitor [21]. An even more advanced set-up would include the use of multichannel NIRS, which has also been described [22].

While BIS monitoring has also become part of standard monitoring, its ability to assess cerebral perfusion during hypothermia with thiopental-induced reduction in cerebral metabolism is limited. Therefore, BIS values during SACP are reduced; however, under conditions of SACP, BIS may be used to indicate the effect of cerebral protection measures reducing cerebral oxygen consumption. In general, to be able to detect brain ischemia with BIS, a constant depth of anesthesia is necessary as an increase in anesthesia depth also decreases BIS values. Nonetheless, current recommendations support the use of processed EEG monitoring in cardiac surgery [5].

We reported previously that multimodal monitoring may increase patient safety in CPB [15]. A multimodal monitoring approach has also been suggested by Zanatta et al. [23]. Azzam et al. recently proposed an algorithm for the use of TCD with cerebral and somatic NIRS [24]. Wang et al. recently reported the use of TCD to determine NIRS thresholds [25]. The actual benefit of such a multimodal approach will have to be addressed by future studies.

## Conclusion

In summary, we suggest that supplemental use of TCD with a B-mode ultrasound device can be useful in patients undergoing aortic arch surgery and may serve to achieve adequate cerebral hemodynamic management during SACP.

## Supplementary Information

**Table **Intraoperative measurements
