# Supplementary material for: Extended neuromonitoring in aortic arch surgery: A case series
Source: Anaesthesist. 2021 Jun 7;70(Suppl 1):68–73. doi: 10.1007/s00101-021-00983-y (PMC8674163; doi:10.1007/s00101-021-00983-y)
Supplement: Supplementary file 1 — Table Intraoperative measurements [file 101_2021_983_MOESM1_ESM.pdf]

Supplemental Material tot he article „Extended Neuromonitoring in Aortic Arch Surgery: A Case Series. Thudium, M.; Kornilov, E.; Hilbert, T et al. (2021) in *Der Anaesthesist.*
Article and supplementary material are available at www.springermedizin.de. Please enter the article title in the search there.

Table Intraoperative Measurements

	Patient number	before CPB	CPB	SACP
**rSO_2 right-sided (%)**	1	75.6	77.5	70.8
	2	66	72.7	44
	3	69	77.3	70.5
	4	79	79.5	95
	5	52	72	63
	6	77	81	61.8
**rSO_2 left-sided (%)**	1	73.8	77.3	71.2
	2	66	73.7	59
	3	72	83.3	63.8
	4	83	85	92
	5	52	77	61
	6	87	89	76.3
**MCAV right-sided (cm/s)**	1	18.4	24	21.4
	2	42	42.1	15.3
	3	64	45.5	34.7
	4	32	33.7	36.7
	5	31.1	61.2	28.7
	6	39	38	12.8
**MCAV left-sided (cm/s)**	1	21.3	21.2	16.2
	2	36	50.5	29
	3	43.5	44.1	24.1
	4	34.2	26	16
	5	82	42.8	41.1
	6	23	20	11.3
**BIS**	1	37	34	2
	2	36	19	0
	3	40	35	18
	4	35	40	16
	5	40	40	3
	6	40	20	6

Data presented as median

CPB cardiopulmonary bypass
SACP selective antegrade cerebral perfusion
NIRS Near infrared spectroscopy
MCAV Middle Cerebral Artery mean flow Velocity
BIS Bispectral Index
